# Characterisation of Australian MRSA Strains ST75- and ST883-MRSA-IV and Analysis of Their Accessory Gene Regulator Locus

**DOI:** 10.1371/journal.pone.0014025

**Published:** 2010-11-17

**Authors:** Stefan Monecke, Hanna Kanig, Wolfram Rudolph, Elke Müller, Geoffrey Coombs, Helmut Hotzel, Peter Slickers, Ralf Ehricht

**Affiliations:** 1 Faculty of Medicine “Carl Gustav Carus”, Institute for Medical Microbiology and Hygiene, Technical University of Dresden, Dresden, Germany; 2 Department for Applied Microbiology, Institute for Microbiology, Technical University of Dresden, Dresden, Germany; 3 Alere Technologies GmbH, Jena, Germany; 4 PathWest Laboratory Medicine-WA, Royal Perth Hospital, Perth, West Australia, Australia; 5 Friedrich-Löffler-Institute, Institute of Bacterial Infections and Zoonoses, Jena, Germany; Cairo University, Egypt

## Abstract

**Background:**

Community-acquired methicillin-resistant *Staphylococcus aureus* have become a major problem in Australia. These strains have now been isolated throughout Australia including remote Indigenous communities that have had minimal exposure to healthcare facilities. Some of these strains, belonging to sequence types ST75 and ST883, have previously been reported to harbour highly divergent alleles of the housekeeping genes used in multilocus sequence typing.

**Methodology/Principal Findings:**

ST75-MRSA-IV and ST883-MRSA-IV isolates were characterised in detail. Morphological features as well as 16S sequences were identical to other *S. aureus* strains. Although a partial *rnpB* gene sequence was not identical to previously known *S. aureus* sequences, it was found to be more closely related to *S. aureus* than to other staphylococci. Isolates also were screened using diagnostic DNA microarrays. These isolates yielded hybridisation results atypical for *S. aureus*. Primer directed amplification assays failed to detect species markers (*femA, katA, sbi*, *spa*). However, arbitrarily primed amplification indicated the presence of unknown alleles of these genes. Isolates could not be assigned to capsule types 1, 5 or 8. The allelic group of the accessory gene regulator (*agr*) locus was not determinable. Sequencing of a region of *agrB, agrC* and *agrD* (approximately 2,100 bp) revealed a divergent sequence. However, this sequence is more related to *S. aureus agr* alleles I and IV than to *agr* sequences from other *Staphylococcus* species. The predicted auto-inducing peptide (AIP) sequence of ST75 was identical to that of *agr* group I, while the predicted AIP sequence of ST883 was identical to *agr* group IV.

**Conclusions/Significance:**

The genetic properties of ST75/ST883-MRSA may be due to a series of evolutionary events in ancient insulated *S. aureus* strains including a convergent evolution leading to *agr* group I- or IV-like AIP sequences and a recent acquisition of SCC*mec* IV elements.

## Introduction

A variety of community-acquired methicillin-resistant *S. aureus* (ca-MRSA) strains with divergent genetic backgrounds have emerged globally. In Australia some of these strains have been isolated from Indigenous people living in remote communities who have had minimal previous exposure to healthcare facilities. One of these strains, ST75-MRSA-IV, is a PVL-negative ca-MRSA frequently isolated from skin and soft tissue infections in people living in the northern regions of Australia [1]. Recently it has been demonstrated that the multilocus sequence typing (MLST) genes in ST75 differ from other *S. aureus*. Using array hybridisation, the accessory gene regulator (*agr*) genes in ST75-MRSA-IV and in a related ST883-MRSA-IV strain could not be detected [2]. Although no phenotypic differences to other *S. aureus* were observed, it has been proposed to classify these strains as a new subspecies of *S. aureus* [3].

As a toxin signatory pathway, the *agr* locus genes encode AgrA, AgrB, AgrC, and AgrD that constitute a virulence regulating quorum-sensing system [4]-[6]. This system responds to the extracellular concentration of a secreted auto-inducing peptide (AIP) derived from AgrD. AgrB is a putative processing enzyme, and AgrC serves as receptor for AIP. Quorum sensing via AIP regulates the expression of RNAIII derived from the *hld* gene. RNAIII up-regulates the production of secreted toxins, but down-regulates genes encoding surface proteins. The central segment of the *agr* locus (the C-terminal two-thirds of AgrB, AgrD, and the N-terminal half of AgrC) shows a striking interstrain variation. Known alleles cluster into four distinct groups [7]. The division of *S. aureus* strains into these *agr* groups is based on their experimentally proven ability to inhibit or stimulate expression of virulence-related genes. The agent mediating this modulation of virulence gene expression is AIP. Its sequence is different in each *agr* group. AIP stimulates expression of virulence genes in its own *agr* group, but represses these genes in strains of a different *agr* group, resulting in bacterial interference [8]. It has previously been proposed that the four different alleles of the *agr* cluster reflect an ancient division in *S. aureus* evolution [7]. However, strains belonging to related clonal complexes based on MLST have been shown to have different *agr* groups. Furthermore, highly divergent clonal complexes have been shown to harbour identical *agr* alleles [2]. Consequently, the concept of a straightforward division of *S. aureus* into four *agr* groups attaining some kind of subspecies status is not valid. Proposals for a phylogenetic tree of *S. aureus*, made to explain the discrepancies between overall genetic background and *agr* group affiliations, assume multiple steps of diversification and recombination [9]; [10].

As ST75-MRSA-IV may be a new subspecies of *S aureus*, the aim of this study was to characterise several of these isolates using phenotypic and genotypic methods. For genotypic characterisation, hybridisation patterns on DNA arrays were analysed. Genes encoding the 16S rRNA and the RNA subunit of RNase P (*rnpB*) genes were sequenced, as these genes have previously been used to infer taxonomy and phylogeny (see, *e.g*., [11]–[14]). As *agr* regulation is a vital pathway in *S. aureus*, the failure to detect known alleles of *agr* locus genes in ST75-MRSA-IV and ST883-MRSA-IV suggests the presence of new alleles of these genes. Consequently, a characterisation of the *agr* locus of these isolates was performed in this study.

## Results

### Phenotypic characterisation

Morphologically on Columbia blood agar, isolates formed yellow-greyish colonies with beta-haemolysis. The clumping factor test was positive. Standard biochemistry was in accordance with the Vitek 2 *S. aureus* profile (Supplemental File S1). The urease test was negative for ST75-MRSA-IV, but positive for ST883-MRSA-IV. ST75-MRSA-IV and ST883-MRSA-IV isolates were also identified as *S. aureus* using MALDI-TOF.

### Array hybridisation

Detailed hybridisation results are provided in Supplemental File S2.

ST75-MRSA-IV and ST883-MRSA-IV provided hybridisation patterns distinct from other *S. aureus* strains. Some of the characteristic *S. aureus* markers including *coa, spa*, *katA, femA* or *sbi* did not yield hybridisation signals in both amplification protocols, or were detectable only when using the sequence-independent random-primer amplification (Supplemental File S2). This can be attributed to the present of distinct sequences which are sufficiently divergent to prevent their detection. Indeed, a previously published ST75 *coa* sequence (GenBank AB436988, strain JCSC1469, [15]) shows mismatches affecting primer and probe binding sites. The *spa* gene is known to be present in both strains, as a *spa* types have previously been reported ([16], 259-31-17-17-17-23-17-17-23-17-22 for ST75-MRSA-IV and 259-23-23-17-17-17-23-23-23-17-16 for ST883-MRSA-IV). For *katA, femA* and *sbi* genes, confirmatory PCRs were designed (Table 1). The ST75-MRSA-IV isolate 03-17848 yielded products of the expected size for these PCRs. The ST883-MRSA-IV isolate 06-16607 was positive in the *femA, sbi* and *katA* (in frame) PCRs, but did not amplify in the *katA* (out frame) PCR.

**Table 1:** Primers used for amplification and sequencing of genes in ST75-MRSA-IV and ST883-MRSA-IV.

PCR	Target localisation	Forward Primer sequences (5′-…-3′)	Reverse Primer sequences (5′-…-3′)	Length (bp)	T_ann_
**Sa16S**	16S rRNA	TTTTATGGAGAGTTTGATCC	AGAAAGGAGGTGATCCAG	1,555	51°C
**Sa16S-1**	Sequencing primer for Sa16S PCR product	GTCCGCCGCTAACATCAG	-	-	55,7°C
**Sa16S-2**	Sequencing primer for Sa16S PCR product	ATAGATGGATCCGCGCTG	-	-	54,1°C
**Sa16S-3**	Sequencing primer for Sa16S PCR product	ACAACCATGCACCACCTG	-	-	55,2°C
**Sa16S-4**	Sequencing primer for Sa16S PCR product	AATCGCTAGTAATCGTAG	-	-	45,7°C
**rnpB-fw-01/rnpB-rv-02**	*rnpB*	AGTTTAACTGTCTTACTAATAATGACT	ACAAGGCAGTGTCATTATATCA	528	60°C
**agrC_uni_fw/agrA_uni_rv**	*agrC* to *agrA*	CATGATTATGTCAATATCTT	TGTTTTCTCTTTGTTTTG	650	33°C
**agrB_uni_fw/agrC_uni_rv**	Upstream of *agrB* to *agrC*	AAAGATTGTACTAAATCGT	GTCGTTAAGATATTGACA	1500	33°C
**pr_agr01_fw/pr_agr02_rv**	*agrB* to *agrC*	ATTTTCTTTATTAAGGAGGA	GAATACCATTTGATTTTGA	435	38°C
**pr_agr03_fw/pr_agr04_rv**	*agrC*	TCAAAATCAAATGGTATTC	TTTTATTGAAATAGTCACG	585	38°C
**pr_agr11-fw/pr_agr12-rv**	*agrB* to *agrD*	GGTGTTATGTAGAGAGTATTATATTGT	AACATTAATATCATTTGAGTTAATACGAA	530	52°C
**pr_agr13-fw/pr_agr14-rv**	*agrC*	CCAAAAGAATTAACTCAATTACACG	AGTAAGCAGTAAGATGGTCGA	530	52°C
**pr_agr15-fw/pr_agr16-rv**	*agrC*	GAAGCAAAAGTAATAAGGCAGT	TCAAGAATAATACCAATACTGCG	530	52°C
**katA_outframe**	*katA*, out frame	CTGATTTTTTAATCATCTTGAGCATG	GTTACGTTTACGCGCACG	1725	55°C
**katA_inframe**	*katA*, in frame	CGAGAAAATAGTATGACAGCAGG	CATAAACTGCTCAACTACGCAATATAG	1505	55°C
**femA_02/04_outframe**	*femA*, out frame	AACGAGAGACAAATAGGAGTAATG	CAAATTCGGTAACAGTTAACTCTG	1345	55°C
**sbi_inframe**	*sbi*, in frame	CAGCAACAATTACTTTAGCTACAATG	CTAGAGAAGATATTTTTGATTGATTATTTCC	1405	55°C

Other species-specific genes such as *gapA*, *nuc, setC, hld*, and some of the genes encoding microbial surface components recognising adhesive matrix molecules (MSCRAMM), such as *bbp, clfA/B, eno, fnbA/B* and *sdrC/D* and the biofilm-associated gene *icaA* were detected. Other MSCRAMM genes, *cna, ebpS, fib, map* and *vwb*, as well as biofilm-associated genes *icaC/D* and *bap* were not detected. The *sasG* gene was detected in ST75, but not in ST883. Isolates did not yield signals with probes for *capH, capI, capJ* and *capK* genes corresponding to the capsule types 1, 5 or 8, in either the primer–directed, or the random amplification protocols.

The affiliation to *agr* groups could not be determined as no signals were obtained with primers and probes derived from all previously described *S. aureus agr* locus sequences. Other regulatory genes, *sarA, saeR, saeS, vraS,* were detectable, while *vraR* yielded weak (in ST75-MRSA-IV) or no signals (in ST883-MRSA-IV).

ST75-MRSA-IV carried the *egc* enterotoxin gene cluster locus including *seg, sei*, *sem, seo* and *seu.* Another gene from this cluster, *sen,* was only detectable by random amplification. The ST75-MRSA-IV isolates differed in the presence of the enterotoxin B gene *seb*. Eleven isolates yielded either no signals, or in the random amplification protocol only, while seven isolates were positive in the primer-directed amplification. Two isolates from the former group failed to produce enterotoxin B, while two isolates from the latter group were positive for toxin production (Supplemental File S3). Enterotoxin genes were not detected in ST883-MRSA-IV, and this strain did not produce enterotoxin B. This isolate also differed from ST75 with regard to some other markers such as *ssl/set* and *hysA* alleles (Supplemental File S2). The gamma haemolysin locus (*hlgA, lukF/S-hlg*), PVL genes (*lukF/S-PV*) and the leukocidin homologues *lukD/E* and “*lukX/Y*” (NC_002951; SACOL2004 and 2005) were not detected.

All isolates carried SCC*mec* IV elements. ST75-MRSA-IV isolates harboured *blaZ, blaR* and *blaI.* Other resistance determinants, *msrA, qacA* and *qacC,* were found in some ST75-MRSA-IV isolates. The ST883-MRSA-IV isolate lacked *blaZ/R/I,* but harboured *ermC.*

### Sequencing

**Figure 1:**
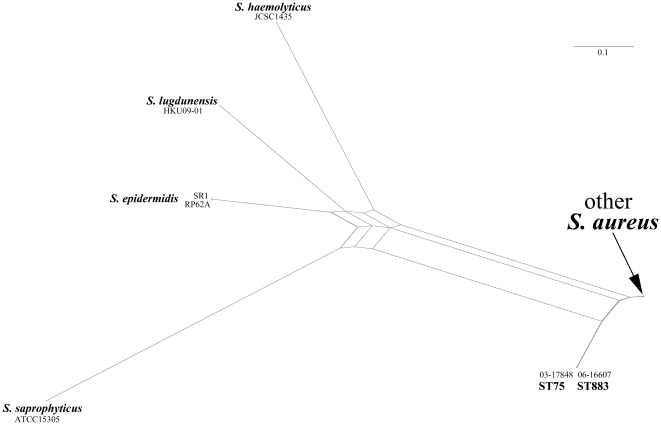
Phylogenetic network reconstruction based on DNA sequences of staphylococcal *rnpB* genes.

The 16S rRNA gene sequences of isolates 03-17848 (ST75-MRSA-IV, HQ260331) and 06-16607 (ST883-MRSA-IV, GenBank HQ260332) were identical to other *S. aureus* strains. The *rnpB* gene sequences of these two strains (GenBank HQ260329 and HQ260330, respectively) were identical to each other. With a 97.5% sequence homology, these two strains were more related to *S. aureus* than to other *Staphylococcus* species (with sequence homologies of 87%; *S. saprophyticus* ATCC 15305, AP008934.1 to 90%; *S. lugdunensis* HKU09-01, CP001837.1; Figure 1).

**Figure 2:**
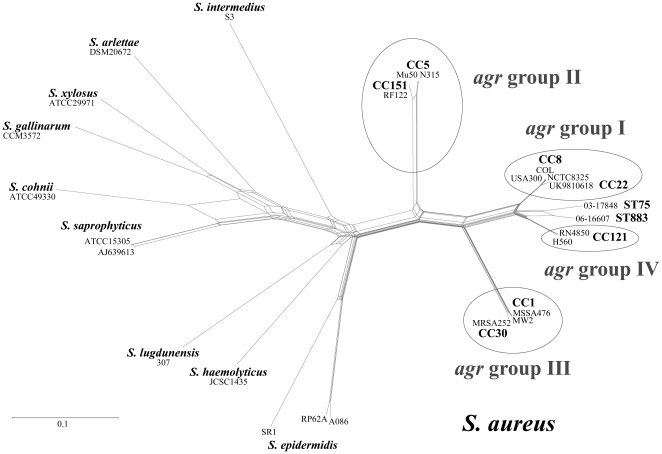
Phylogenetic network reconstruction based on DNA sequences of staphylococcal *agr* genes.

Approximately 2,045 base pairs of *agrB, agrD* and *agrC* of the ST75-MRSA-IV isolate 03-17848 were sequenced (GenBank FJ154839). Sequences revealed differences to known *agr S. aureus* sequences. Although they were not completely identical, *agrB, agrD* and *agrC* sequences from the ST883-MRSA-IV isolate 06-16607 (GenBank HQ260328) resembled ST75-MRSA-IV. BLAST yielded an identity of 92% with query coverage of 100%. Both sequences are more closely related to *S. aureus agr* alleles than to other staphylococcal *agr* sequences (Figure 2). Among the *S. aureus agr* alleles, they are more closely related to *agr* group I and IV than to *agr* groups II or III. A protein sequence alignment (Supplemental File S4) revealed that the putative AIP sequence of ST75 was identical to *agr* group I, and that the predicted AIP sequence of ST883 was identical to *agr* group IV.

## Discussion

Australian ca-MRSA strains characterised as ST75-MRSA-IV and ST883-MRSA-IV have previously been shown to have MLST alleles that are unique compared to other *S. aureus* genotypes (see http://saureus.mlst.net/). In this study we have shown that the array hybridisation patterns and the *agr* and *rnpB* gene sequences for these two strains also differ from other *S. aureus* strains. Furthermore, species-specific genes such as *femA, coa, sbi* or *spa* and capsule genes were either absent or their sequences were sufficiently divergent in the isolates studied to prevent their detection by DNA array hybridisation. Whether these differences justify the recognition of ST75 and ST883 as another species or as distinct subspecies [3] is a matter of debate. Of practical consequence is that nucleic acid based tests in general may not yield a correct result because their performance depends on the underlying sequence information. New variants or alleles of target genes may not be detected if sequences of probe or primer binding sites deviate from known sequences on which the design of the test relied. Thus, our DNA array results may indicate that known genome sequences do not reflect the whole biodiversity of *S. aureus*, and consequently whole genome sequencing of divergent strains such as ST75-MRSA-IV or ST883-MRSA-IV would be helpful.

While ST75-MRSA-IV are different from other *S. aureus*, the isolates from this strain are rather homogenous. Variability however was detected in the presence of *seb* gene and, correspondingly, in the production of enterotoxin B. Only isolates, in which *seb* was detected by primer-directed amplification were shown to produce enterotoxin B. Complete or partial absence of the *seb* gene or its replacement by a geno- and phenotypically divergent variant are likely explanations for this observation. The only other variations observed in ST75-MRSA-IV affected possibly mobile resistance markers. Although ST883-MRSA-IV lacks *seb* as well as the enterotoxin gene cluster *egc* and differs in some MLST alleles (see http://saureus.mlst.net/), this strain shares, in terms of hybridisation profile and *rnpB* gene sequences, some features with ST75-MRSA-IV (see above and Supplemental File S2).

The *agr* sequences of ST75 and ST883 are distinct from previously known *agr* sequences. Furthermore, an 8% difference between the *agr* sequences of these two strains was identified. For comparison, a fragment of identical length from *agr* II sequences of Mu50/N315 (CC5) and RF122 (CC151) differ by only 4%.

Based on DNA sequence, ST75/ST883 *agr* alleles could be considered as representatives of a novel *agr* group although they are less uniform than the other *agr* alleles. However, *agr* groups are conventionally defined by phenotype rather than by genotype. Although it was beyond the scope of this study to test ST75/ST883 for bacterial interference with other *S. aureus* strains or to determine the amino acid sequence of the mature AIP from ST75 and ST883, predicted AIP amino acid sequences derived from gene sequences were analysed. Predicted AIP amino acid sequence differed for ST75 and ST883. ST75 yielded an *agr* group I AIP amino acid sequence, while the AIP amino acid sequence of ST883 was identical to *agr* group IV (Supplemental File S4). For this reason, despite the differences demonstrated in the *agr* gene sequences, these strains cannot be regarded as prototypic for a novel *agr* group.

The *agr* alleles I to IV are distributed world-wide. The *agr* groups I to III are known to include a variety of pandemic MRSA and MSSA strains. The *agr* allele IV is less diverse, found only in a few *S aureus* strains including ST50 and ST121 [9]. Although no major MRSA strains belong to *agr* group IV, MSSA of ST121 are known to be common and widespread in several countries [17]–[20]. In contrast, strains with ST75/ST883-like *agr* allele*s* have only been found in remote regions of northern Australia, where ST75-MRSA-IV is frequently isolated [1]. Although no data on methicillin-susceptible precursor strains are available, we assume ST75- and ST883-MRSA strains originated in northern Australia, and have evolved from pre-existent, local MSSA strains by acquisition of SCC*mec* elements. While their genetic background differs from other *S. aureus* strains, ST75- and ST883-MRSA strains harbour SCC*mec* IV elements suggesting a recent acquisition of these elements. Thus we assume that ST75/ST883 may have evolved independently from other *S. aureus* populations over an extended period of time and in recent years has been transformed to MRSA by the global “infection-like” spread of SCC*mec* elements affecting several staphylococcal species.

The genetic differences in ST75/ST883 may be due to a series of evolutionary events in an ancient, insulated *S. aureus* strain. *S. aureus* is a well adapted human parasite, which may have co-evolved with humans resulting in a prehistoric radiation of *S. aureus* into a number of divergent *agr* alleles. Much later, in the ages of discovery and colonisation, a few *S. aureus* clonal lineages of European, East Asian or African origin may have been spread worldwide. For example, a recently published analysis of sequence polymorphisms in ST5 (a global sequence type belonging to *agr* group II) suggests a higher degree of diversity in Europe, and an expansion approximately 1,000 years ago [21]. The *agr-*mediated bacterial interference [8] could have been a driving force for an evolution towards a very small number of competing allelic variants (I to IV) resulting either in extinction of other *agr* alleles, or in convergent evolution leading to similar AIP and *agr* gene sequences. Evolution towards a certain uniformity might be a stable strategy for competitors facing the same selective pressure, as economists have noted already long ago [22]. Ancient, native *S. aureus* strains may have been able to survive due to convergent evolution or to recombination events resulting in the acquisition of one of the four globally predominant *agr* alleles. We observed that predicted AIP amino acid sequences of ST75 and ST883 are identical to AIP sequences of *agr* group I and IV, respectively, although their genetic background including the rest of their *agr* sequences is different. This could indicate a convergent evolution towards a limited number of main *agr* types. *S aureus* of ST93 (including another unique Australian caMRSA, the Queensland Clone, ST93-MRSA-IV, [23]) may be another example. Its overall genetic background also differs from the currently known sequences resulting in a highly divergent hybridisation profile in microarray experiments [2]. Although in this regard it resembles ST75 and ST883, ST93 harbours an inconspicuous *agr* type III locus [2]; [24].

In order to prove this concept of *S. aureus* evolution, historical isolates and isolates from remote human communities and/or native populations in different parts of the world should be collected and scrutinised. This could provide an insight into the evolution and biodiversity of an important human pathogen.

## Materials and Methods

### Strains and isolates

ST75-MRSA-IV: Eighteen isolates were collected between 2003 and 2008. Thirteen originated from the northern region of Western Australia (03-17848, 04-15040, 04-15225, 04-15678, 04-15778, 04-16031, 04-16785, 04-17342, 04-17542, 05-15398, 05-15944, 06-16850, 08-18362), four from the Northern Territory (03RDH76, 03RDH78, 03RDH93, 04RDH34) and one from Queensland (06RBH23). Isolates were obtained from skin and soft tissue infections or from screening cultures. All were genotyped by DNA-array hybridisation. Phenotypic characterisation was performed on five isolates (03-17848, 04-16031, 04-16785, 03RDH76, 06RBH23) using Vitek-2 and MALDI-TOF. Four isolates (03-17848, 04-16785, 04-17542, 05-15398) were screened for enterotoxin B production. One isolate (03-17848) was selected for further characterisation and sequencing.

ST883-MRSA-IV: A single isolate (06-16607) was characterised which was obtained from a hand swab collected in 2006 in the northern region of Western Australia.

No patient data were stored or used for this study; thus formal ethics committee approval and informed consent from the patients were not required.

Control strains: Fully sequenced strains MSSA476 (ST1-MSSA), MW2-USA400 (ST1-MRSA-IV), Mu50 (ST5-MRSA-II), N315 (ST5-MRSA-II), NCTC 8325 (ST8-MSSA), COL (ST250-MRSA-I), USA300-FPR3757 (ST8-MRSA-IV), and Sanger MRSA 252 (CC30-MRSA-II) were included for comparison.

### Phenotypic characterisation

Culture morphology was assessed on Columbia blood agar. The clumping factor test was performed using a commercial assay (Pastorex Staph-Plus, Bio-Rad, Munich, Germany). Standard biochemistry was determined by the Vitek 2 Gram Positive Identification card (BioMerieux, Nuertingen, Germany). MALDI-TOF analysis was performed using the MALDI Biotyper (Bruker, Bremen, Germany) according to manufacturer's specifications.

### Pulsed field gel electrophoresis, *spa-*, MLST and SCC*mec*-typing

Pulsed field gel electrophoresis, *spa-*, and MLST typing procedures were performed according to previously published protocols [25]–[27]. SCC*mec*-typing procedures were performed as described previously [28].

### Array procedures

The DNA array (StaphyType system by Alere Technologies GmbH, Jena, Germany) used in this study included probes for 334 different target sequences which corresponds to, depending on the nomenclature used, approximately 180 distinct genes and their different alleles. This included species controls, typing markers (*agr*, capsule and SCC*mec*-related genes), genes encoding exotoxins or MSCRAMMs as well as resistance markers.

After overnight incubation on Columbia blood agar, colonies were harvested and lysed using lysostaphin, lysozyme and ribonuclease A followed by digestion with proteinase K. The DNA was purified using the QIAGEN device EZ1 (QIAGEN, Hilden, Germany) according to the manufacturer's tissue lysis protocol.

Two different protocols were used for linear amplification and biotin labelling of amplicons. The first, a target sequence-dependent, primer-directed amplification protocol, [2]; [24]; [29]; [30] is a thermally synchronised iterated linear primer elongation method in which a mixture of antisense primers for all targets facilitated a simultaneous copying of all target sequences to single stranded DNA. Within this step, amplicons are labelled by incorporation of biotin-16-dUTP.

The second protocol is a target sequence-independent random amplification procedure [31]–[34] resulting in biotin labelled amplicons representing the complete input genome sequence. This method detects target genes for which the binding sites of the primers used in the first protocol were deleted or changed by nucleotide polymorphisms. Thus, this method is more suited to characterise strains for which sequence data are not available yet. Primers were used which consisted of a random sequence and of an invariant part. The latter was used as a primer binding site for a second round of amplification. Amplicons were also labelled by incorporation of biotin-16-dUTP.

Post amplification, the labelled samples were denatured and hybridised to the array. This was followed by several washing steps and the addition of a blocking reagent. Horseradish-peroxidase-streptavidin conjugate was added to the array, followed by incubation and washing. A precipitating dye (Seramun Green, Seramun, Heidesee, Germany) was then added, and the hybridisation pattern was analysed using a designated reading device and software (ArrayMate by Alere Technologies GmbH).

### Sequence analysis of 16S rRNA genes

16S rRNA genes were amplified using Sa16S-F (5′-TTT TAT GGA GAG TTT GAT CC-3′) and Sa16S-R (5′-AGA AAG GAG GTG ATC CAG-3′) primers. The resulting PCR products (1,555 bp) were purified and sequenced with amplification primers and Sa16S-1-4 primers (Table 1). 20 ng of purified DNA and the BigDye Terminator v1.1 Cycle Sequencing Kit (Applied Biosystems) were used in cycle sequencing according to manufacturer's instructions.

### Sequence analysis of *rnpB*

The *rnpB* locus was amplified using genomic DNA and rnpB-fw-01 (5′- AGTTTAACTGTCTTACTAATAATGACT-3′) and rnpB-rev-02 (5′- ACAAGGCAGTGTCATTATATCA-3′) primers. The resulting PCR products (528 bp) were subcloned using the TOPO TA Cloning Kit (Invitrogen, Darmstadt, Germany). Sequencing of purified plasmid DNA was carried out as a cycle sequencing procedure using the BigDye Terminator v1.1 Cycle Sequencing Kit (Applied Biosystems). Briefly, 200 ng of plasmid DNA, M13 reverse primer (5′-CAG GAA ACA GCT ATG AC-3′) and M13 (-21) primer (5′-GTA AAA CGA CGG CCA GT-3′) were used in this process. Sequencing products were analysed on an ABI Prism 310 Genetic Analyzer (Applied Biosystems).

### Sequence analysis of the *agr* locus

Primers used for the sequencing of the ST75 and ST883 *agr* allele are listed in Table 1. Primers agrC_uni_fw, agrA_uni_rv, agrB_uni_fw and agrC_uni_rv were derived from conserved loci of known *agr* alleles. Primers were also derived from sequences obtained by the use of agrC_uni_fw, agrA_uni_rv, agrB_uni_fw and agrC_uni_rv primers. DNA sequencing was performed using either PCR products obtained with agrA_uni_rv and agrB_uni_fw primers or purified plasmid DNA resulting from cloning these PCR products. This was performed as a cycle sequencing procedure using BigDye Terminator v1.1 Cycle Sequencing Kit (Applied Biosystems, Darmstadt, Germany) according to the manufacturer's recommendations as well as DNA sequencers ABI Prism 377 or 3100 (Perkin-Elmer/Applied Biosystems, Foster City, CA).

### Enterotoxin B detection

Protein arrays using enterotoxin B specific capture and detection antibodies were used to test four ST75-MRSA-IV isolates and the ST883-MRSA-IV-isolate for the production of enterotoxin B. Culture supernatants (overnight incubation, liquid medium as described by Kato and Noda [35]) were used for testing in a 1∶10 dilution. Details on technology and protocols have been described previously [36].

### Phylogenetic tree reconstruction

For phylogenetic tree reconstruction, SplitsTree software [37] was used with default settings (characters transformation, uncorrected P; distance transformation, Neighbour-Net; and variance, ordinary least squares).

For Figure 1, staphylococcal *rnpB* genes were analysed. Beside the ST75 and ST883 sequences obtained in this study, the following GenBank entries were used: “other *S. aureus*”, *i.e.*, MSSA476, BX571857.1; MW2, BA000033.2; Mu50, BA000017.4; N315, BA000018.3; COL, CP000046.1; NCTC8325, CP000253.1; USA300-FPR3757, CP000255 and MRSA252, BX571856.1 as well as *S. epidermidis* RP62A, CP000029.1 and SR1, AF270230.1; *S. haemolyticus* JCSC1435, AP006716.1; *S*. *lugdunensis* HKU09-01, CP001837.1; *S. saprophyticus* ATCC15305, AP008934.1.

For Figure 2, staphylococcal *agr* genes were analysed. Beside the ST75 and ST883 sequences from this study, the following GenBank entries were used*:* MSSA476, BX571857.1; MW2, BA000033.2; Mu50, BA000017.4; N315, BA000018.3; COL, CP000046.1; NCTC8325, CP000253.1; USA300-FPR3757, CP000255; H560, DQ157981.1; RN4850, DQ229853.1; RF122, AJ938182.1; UK9810618, DQ157966 and MRSA252, BX571856.1; *S. arlettae* DSM20672, AF346712.1; *S. cohnii* ATCC49330, AF346721.1; *S. epidermidis* A086, Z49220.1 as well as RP62A, CP000029.1 and SR1, AF270230.1; *S. gallinarum* CCM3572, AF346722.; *S. haemolyticus* JCSC1435, AP006716.1; *S. intermedius* S3, AY965912.1; *S*. *lugdunensis* 307, AF173933.1; *S. saprophyticus* ATCC15305, AP008934.1 and AJ639613.1 as well as *S. xylosus* ATCC29971, AF346729.1.

## Supporting Information

File S1 This file shows the Vitek 2 biochemical profile of the study strains in comparison to a reference strain.
(0.01 MB PDF)

File S2 This files shows array hybridisation results of ST75-MRSA-IV and ST883-MRSA-IV as well as of some reference strains for comparison.
(1.19 MB PDF)

File S3 This files shows the detection of enterotoxin B in studied strains and reference strains.
(2.78 MB PDF)

File S4 This file shows that, despite of unique *agr* gene sequences, predicted autoinducing peptide sequences of ST75 and ST883 allow them to be assigned to *agr* groups I and IV, respectively.
(0.01 MB PDF)
