# Supplementary material for: Characterisation of Australian MRSA Strains ST75- and ST883-MRSA-IV and Analysis of Their Accessory Gene Regulator Locus
Source: PLoS One. 2010 Nov 17;5(11):e14025. doi: 10.1371/journal.pone.0014025 (PMC2984443; doi:10.1371/journal.pone.0014025)
Supplement: File S1 — This file shows the Vitek 2 biochemical profile of the study strains in comparison to a reference strain. (0.01 MB PDF) [file pone.0014025.s001.pdf]

Supplemental File S1: Vitek 2 profiles of ST75-MRSA-IV and ST883-MRSA-IV

For comparison, data of ATCC29213 (the quality control strain as recommended by the manufacturer) are also displayed.

**Substrate**		***S. aureus* ATCC29213**	**ST75-MRSA-IV (5 isolates)**	**ST883-MRSA-IV (1 isolate)**
Arginine dihydrolase	ADH1	POSITIVE	POSITIVE	POSITIVE
Arginine dihydrolase	ADH2S	VARIABLE	NEGATIVE	POSITIVE
Alpha -galactosidase	AGAL	NEGATIVE	NEGATIVE	NEGATIVE
Alpha-glucosidase	AGLU	VARIABLE	VARIABLE (3 out of 5 pos.)	NEGATIVE
Alanine arylamidase	ALAA	NEGATIVE	NEGATIVE	NEGATIVE
Alpha-mannosidase	AMAN	NEGATIVE	NEGATIVE	NEGATIVE
Amygdalin	AMY	NEGATIVE	NEGATIVE	NEGATIVE
Ala-phe-pro arylamidase	APPA	NEGATIVE	NEGATIVE	NEGATIVE
L-aspartic acid arylamidase	ASPA	NEGATIVE	NEGATIVE	NEGATIVE
Bacitracin resistance	BACI	POSITIVE	VARIABLE (4 out of 5 pos.)	POSITIVE
Beta-galactosidase	BGAL	VARIABLE	NEGATIVE	NEGATIVE
Beta-Glucorinidase	BGUR	NEGATIVE	NEGATIVE	NEGATIVE
Beta-Glucorinidase	BGURR	NEGATIVE	NEGATIVE	NEGATIVE
alpha-cyclodextrin	CDEX	NEGATIVE	NEGATIVE	NEGATIVE
Galactose	DGAL	VARIABLE	VARIABLE (2 out of 5 pos.)	POSITIVE
Maltose	DMAL	POSITIVE	POSITIVE	POSITIVE
Mannitol	DMAN	POSITIVE	POSITIVE	POSITIVE
Mannose	DMNE	POSITIVE	POSITIVE	POSITIVE
Raffinose	DRAF	NEGATIVE	NEGATIVE	NEGATIVE
Ribose	DRIB	VARIABLE	VARIABLE (1 out of 5 pos.)	NEGATIVE
Sorbitol	DSOR	NEGATIVE	NEGATIVE	NEGATIVE
Trehalose	DTRE	NEGATIVE	POSITIVE	POSITIVE
Xylose	DXYL	NEGATIVE	NEGATIVE	NEGATIVE
Lactose	LAC	VARIABLE	NEGATIVE	NEGATIVE
L-leucine arylamidase	LEUA	VARIABLE	NEGATIVE	NEGATIVE
Lactate	LLATK	POSITIVE	VARIABLE (4 out of 5 pos.)	POSITIVE
Methyl-beta-D-glucopyranoside	MBDG	POSITIVE	POSITIVE	POSITIVE
N-acetyl-glucosamine	NAG	VARIABLE	VARIABLE (3 out of 5 pos.)	NEGATIVE
Growth in 6.5 NaCl	NC6.5	POSITIVE	POSITIVE	POSITIVE
Novobiocin resistance	NOVO	VARIABLE	NEGATIVE	NEGATIVE
O/129 resistance	O129R	POSITIVE	VARIABLE (4 out of 5 pos.)	POSITIVE
Optochin resistance	OPTO	POSITIVE	POSITIVE	POSITIVE
Alkaline phosphatase	PHOS	POSITIVE	POSITIVE	POSITIVE
Phosphatidylinositol-Phospholipase C	PIPLC	NEGATIVE	NEGATIVE	NEGATIVE
Polymyxin B resistance	POLYB	VARIABLE	VARIABLE (4 out of 5 pos.)	POSITIVE
Pro arylamidase beta-glucuronidase	PROA	NEGATIVE	NEGATIVE	NEGATIVE
Pullulan	PUL	NEGATIVE	NEGATIVE	NEGATIVE
L-pyroglutamic acid arylamidase	PYRA	POSITIVE	POSITIVE	NEGATIVE
Sucrose	SAC	POSITIVE	POSITIVE	POSITIVE
Salicin	SAL	NEGATIVE	NEGATIVE	NEGATIVE
Tyrosine arylamidase	TYRA	NEGATIVE	NEGATIVE	NEGATIVE
Urease	URE	NEGATIVE	NEGATIVE	POSITIVE
