# Supplementary material for: Characterisation of Australian MRSA Strains ST75- and ST883-MRSA-IV and Analysis of Their Accessory Gene Regulator Locus
Source: PLoS One. 2010 Nov 17;5(11):e14025. doi: 10.1371/journal.pone.0014025 (PMC2984443; doi:10.1371/journal.pone.0014025)
Supplement: File S2 — This files shows array hybridisation results of ST75-MRSA-IV and ST883-MRSA-IV as well as of some reference strains for comparison. (1.19 MB PDF) [file pone.0014025.s002.pdf]

Supplemental File S2: Array hybridisation results for ST75-MRSA-IV and ST883-MRSA-IV. For comparison, hybridisation profiles of some sequenced reference strains are also displayed. For details on target genes, probes and nomenclature, see [2]. Data on variable genes are provided in the text.

Species markers, regulatory genes and agr	Ribos. STAU	femA	gapA	katA	coA	nuc1	Protein A	sbi	sarA	saeR	saeS	vraR	vraS	vraS	agrI	agrII	agrIII	agrIV	"agrV" (PCR)	hld
NCTC 8325	●	●	●	●	●	●	●	●	●	●	●	●	●	●	●					●
COL	●	●	●	●	●	●	●	●	●	●	●	●	●	●	●					●
USA300-FPR3757	●	●	●	●	●	●	●	●	●	●	●	●	●	●	●					●
MU50	●	●	●	●	●	●	●	●	●	●	●	●	●	●		●				●
N315	●	●	●	●	●	●	●	●	●	●	●	●	●	●		●				●
Sanger MSSA 476	●	●	●	●	●	●	●	●	●	●	●	●	●	●			●			●
MW2	●	●	●	●	●	●	●	●	●	●	●	●	●	●			●			●
Sanger MRSA 252	●	●	●	●	●	●	●	●	●	●	●	●	●	●			●			●
ST75-MRSA-IV	●	○	●	○		●	○	○	●	●	○	○	●	●					●	●
ST883-MRSA-IV	●		●	○		○	○	○	●	○	○		○	●					●	●

SCCmec-associated genes	mecA	delta_mecR	ugpQ	ccrA-1	ccrB-1	plsSCC-COL	Q9XB68-dcs	ccrA-2	ccrB-2	kdpA-E	mecI	mecR	xylR	ccrA-3	ccrB-3	merA-T	ccrAA-85-2082	ccrAA-MRSAZH47	ccrC-85-2082	ccrA-4	ccrB-4
NCTC 8325																					
COL	●	●	●	●	●	●	●														
USA300-FPR3757	●	●	●				●	●	●												
MU50	●	●	●				●	●	●	●	●	●	●								
N315	●	●	●				●	●	●	●	●	●	●								
Sanger MSSA 476				●	●																
MW2	●	●	●				●	●	●												
Sanger MRSA 252	●	●	●				●	●	●	●	●	●	●								
ST75-MRSA-IV	●	●	●				●	●	●												
ST883-MRSA-IV	●	●	●				○	●	●												

Resistance genes I	blaZ	blaI	blaR	ermA	ermC	linA	msrA	mefA	mpbBM	vatA	vatB	vga	vgaA	vgb	aacA-aphD	aadD	aphA_3	sat
NCTC 8325																		
COL																		
USA300-FPR3757					●													
MU50				●											●	●		
N315	●	●	●	●												●		
Sanger MSSA 476	●	●	●															
MW2	●	●	●															
Sanger MRSA 252	●	●	●	●												●		
ST75-MRSA-IV	●	●	●				◑											
ST883-MRSA-IV					●													

● = positive in both amplification protocols ◑ = variable ○ = positive in random amplification only

Resistance genes II	dfrA	far1	Q6GD50 (fusidic acid resist.)	mupR	tetK	tetM	tetEfflux	cat-pC221	cfr	fexA	fosB	qacA	qacC	vanA	vanB	vanZ
NCTC 8325							●				●					
COL					●		●				●					
USA300-FPR3757				●	●		●				●					
MU50						●	●				●	●				
N315							●				●					
Sanger MSSA 476			●				●									
MW2							●									
Sanger MRSA 252							●				●					
ST75-MRSA-IV											●	◐	◐			
ST883-MRSA-IV																

Superantigenic Toxins	tst	sea	sea-320E	sea-N315 (aka entP)	seb	sec/sel	sed/sej/ser	seh	see	sek/seq	seg	sei	sem	sen	seo	seu	Enterotoxin homologue	ORF CM14
NCTC 8325																	●	
COL					●					●							●	
USA300-FPR3757										●							●	
MU50	●	●				●					●	●	●	●	●	●	●	
N315	●			●		●					●	●	●	●	●	●	●	
Sanger MSSA 476		●						●		●							●	
MW2		●				●		●		●							●	
Sanger MRSA 252		●									●	●	●	●	●	●	●	
ST75-MRSA-IV					◐						●	●	●	○	●	●		
ST883-MRSA-IV																		

Leukocidins and haemolysins	lukF	lukS	lukS-ST22+ST45	hlgA	lukF/S-PV	lukF-P83/M	lukD/E	lukX	lukY_var1	lukY_var2	hl	hla	hlIII-all	hlb	un-truncated hlb	sak	chp	scn
NCTC 8325	●	●		●			●	●	●		●	●	●	●		●	●	●
COL	●	●		●			●	●	●		●	●	●	●	●			
USA300-FPR3757	●	●	○	●	●		●	●	●		●	●	●	●		●	●	●
MU50	●	●		●			●	●	●		●	●	●	●		●		●
N315	●	●		●			●	●	●		●	●	●	●		●	●	●
Sanger MSSA 476	●	●		●			●	●	●		●	●	●	●		●		●
MW2	●	●		●	●		●	●	●		●	●	●	●		●		●
Sanger MRSA 252	●	●		●				●		●	●	●	●	○		●	●	●
ST75-MRSA-IV												○	○	○		●		●
ST883-MRSA-IV														○				

● = positive in both amplification protocols ◐ = variable ○ = positive in random amplification only

Misc. virulence factors and proteases	etA	etB	etD	edinA	edinB	edinC	aur_All	aur-OtherThan252	aur-MRSA252	splA	splB	splE	sspA-all	sspB	sspP
NCTC 8325							●	●		●	●	●	●	●	●
COL							●	●		●	●	●	●	●	●
USA300-FPR3757							●	●		●	●	●	●	●	●
MU50							●	●		●	●		●	●	●
N315							●	●		●	●		●	●	●
Sanger MSSA 476							●	●		●	●		●	●	●
MW2							●	●		●	●		●	●	●
Sanger MRSA 252							●		●			●	●	●	●
ST75-MRSA-IV							○	○					○	○	
ST883-MRSA-IV							○	○					○	○	

ssl/set-genes I	setC_MW0345	setB3	setB3 (MRSA252)	setB2	setB2 (MRSA252)	setB1	setB1 (MRSA252)	set6-var1_11	set6-var2_11	set6-var1_12	set6-var2_12	set6-var4_11	ssl01- RF122	ssl2 / set7	ssl2 / set7 (MRSA252)	ssl3 / set8	ssl3 / set8 (MRSA252, SAR0424)
NCTC 8325	●	●		●		●	○	●		○		●		●		●	
COL	●	●		●		●	○	●		○		●		●		●	
USA300-FPR3757	●	●		●		●	○	●		○		●		●	○	●	
MU50	●	●		●		●		●		●		●		●		●	
N315	●	●		●		●		●		●		●		●		●	
Sanger MSSA 476	●	●		●		●			●		●			●		●	
MW2	●	●		●		●	○		●		●			●		●	
Sanger MRSA 252			●		●	○	●		●	●		●			●		●
ST75-MRSA-IV	●					○					●						
ST883-MRSA-IV	●								○		○		○				

ssl/set-genes II	ssl4 / set9	ssl4 / set9 (MRSA252, SAR0425)	ssl05	ssl5 / set3 (MRSA252)	ssl06-NCTC+MW2	ssl7 / set1	ssl7 / set1 (MRSA252)	ssl08	ssl09	ssl9 / set5 (MRSA252)	ssl10 / set4	ssl10 (RF122)	ssl10 / set4 (MRSA252)	ssl11 / set2 (COL)	ssl11 / set2(Mu50/N315)
NCTC 8325	●		●		●	●	○	●	●		●			●	
COL	●								●		●			●	
USA300-FPR3757	●		●		●	●	○	●	●		●			●	
MU50			●			●		●	●		●				●
N315	●		●			●		●	●		●				●
Sanger MSSA 476	●		●		●	●		●	●		●				
MW2	●		●		●	●		●	●		●				
Sanger MRSA 252		●		●			●			●			●		
ST75-MRSA-IV		○				●	○				○		○		
ST883-MRSA-IV	○	○			●	○		●	●		●		○		○

● = positive in both amplification protocols

○ = positive in random amplification only

Capsule and biofilm genes	capH1	capI1	capJ1	capK1	capH5	capI5	capJ5	capK5	capH8	capI8	capJ8	capK8	icaA	icaC	icaD	bap
NCTC 8325					●	●	●	●					●	●	●	
COL					●	●	●	●					●	●	●	
USA300-FPR3757					●	●	●	●					●	●	●	
MU50					●	●	●	●					●	●	●	
N315					●	●	●	●					●	●	●	
Sanger MSSA 476									●	●	●	●	●	●	●	
MW2									●	●	●	●	●	●	●	
Sanger MRSA 252									●	●	●	●	●	●	●	
ST75-MRSA-IV													●			
ST883-MRSA-IV													○			

MSCRAMM genes I	bbp-all	bbp-COL+MW2	bbp-MRSA252	bbp-Mu50	bbp-MW2	bbp-RF122	bbp-ST45	clfA-all	clfA-COL+RF122	clfA-MRSA252	clfA-Mu50+MW2	clfB-all	clfB-COL+Mu50	clfB-MW2	clfB-RF122	cna
NCTC 8325								●	●		●	●	●			
COL	●	●						●	●		●	●	●			
USA300-FPR3757	●	●						●	●	●	●	●	●			
MU50	●			●				●			●	●	●			
N315	●			●				●			●	●	●			
Sanger MSSA 476	●	●			●			●			●	●		●	○	●
MW2	●	●			●			●			●	●		●	●	●
Sanger MRSA 252	●		●					●		●		●				●
ST75-MRSA-IV	●		○		●					●		○	●			
ST883-MRSA-IV	●		●		○		○	○		●		●	●			

MSCRAMM genes II	ebh-all	ebh-COL	ebh-COL+Mu50	ebh-MW2+MRSA252	ebpS	ebpS-COL	eno	fib	fib-MRSA252	fnbA-all	fnbA-COL	fnbA-MRSA252	fnbA-Mu50+MW2	fnbA-RF122	fnbB-COL	fnbB-COL+Mu50+MW2	fnbB-CC5,15,30,45	fnbB-MW2	fnbB-ST15	fnbB-ST45-2
NCTC 8325	●	●	●		●	●	●	●		●	●				●	●				
COL	●	●	●		●	●	●	●		●	●				●	●				
USA300-FPR3757	●	●	●		●	●	●	●		●	●				●	●	●			
MU50	●		●		●		●	●		●			●			●	●			
N315	●		●		●		●	●		●			●			●	●			
Sanger MSSA 476	●			●	●		●	●		●			●			○		●		
MW2	●			●	●		●	●		●			●			●		●		
Sanger MRSA 252	●			●	●		●		●	●		●								
ST75-MRSA-IV							●			●				●			●		●	
ST883-MRSA-IV			○				●			●						○	●		○	

● = positive in both amplification protocols

○ = positive in random amplification only

MSCRAMM genes III	map-COL	map-MRSA252	map-Mu50+MW2	sasG-COL+Mu50	sasG-MW2	sasG-Other than MRSA252 & RF122	sdrC-all	sdrC-COL	sdrC-Mu50	sdrC-MW2+MRSA252+RF122	sdrD-all	sdrD-COL+MW2	sdrD-Mu50	sdrD-other1	vwb-all	vwb-COL+MW2	vwb-MRSA252	vwb-Mu50	vwb-RF122
NCTC 8325	●			●		●	●	●			●	●			●	●			
COL	●			●		●	●	●			●	●			●	●			
USA300-FPR3757	●		○	●		●	●	●			●	●			●	●			
MU50	○		●	●		●	●		●		●		●		●			●	
N315	○		●	●		●	●		●		●		●		●			●	
Sanger MSSA 476			●		●	●	●			●	●	●			●	●			
MW2	○		○		●	●	●			●	●	●			●	●			
Sanger MRSA 252		●					●			●					●		●		
ST75-MRSA-IV					●		●				○			●					
ST883-MRSA-IV							●		●	○	●			●	●				

Misc. genes I	isaB	mprF-COL+MW2	mprF-Mu50+252	isdA	isdA-Other than MRSA252	lmrP-Other than RF122	lmrP-RF122	hsdS1-RF122	hsdS2-ST5+ST8	hsdS2-MW2+476	hsdS2-RF122	hsdS2-MRSA252	hsdS3- Other than MRSA252 & RF122	hsdS3-ST8+ST1+RF122	hsdS3-Mu50+N315	hsdS3-MRSA252	hsdSx-CC25	hsdSx-CC15	hsdSx-etd
NCTC 8325	●	●		●	●	●			●				●	●			●		
COL	●	●		●	●	●			●				●	●			●		
USA300-FPR3757	●	●	○	●	●	●			●				●	●			●		
MU50	●		●	●	●	●			●				●		●		●		
N315	●		●	●	●	●			●				●		●		●		
Sanger MSSA 476	●	●		●	●	●				●			●	●			●		
MW2	●	●		●	●	●				●			●	●			●		
Sanger MRSA 252			●	●		●						●				●			
ST75-MRSA-IV		●	○			○					●						○		
ST883-MRSA-IV	○	●	○			●		●	●				○				○		

Misc. genes II	Q2YUB3	Q2FXC0	Q2G2T0	Q7A4X2	Q9XB68-MSSA476	yujE	yxeA	hysA1-MRSA252	hysA1-MRSA252+RF122 and hysA2-all	hysA1-MRSA252+RF122 and hysA2-COL+USA300	hysA2- Other than MRSA252	hysA2-COL+USA300+NCTC	hysA2- Other than COL+USA300+NCTC	hysA2-Other than COL+USA300+NCTC	hysA2-MRSA252
NCTC 8325		●	●			●	●		●	●	●	●			
COL		●	●			●	●		●	●	●	●			
USA300-FPR3757		●	●		○	●	●		●	●	●	●			
MU50			●	●		●	●		●		●		●	●	
N315			●	●		●	●		●		●		●	●	
Sanger MSSA 476		●	●		●	●	●		●		●		●	●	
MW2		●	●		●	●	●		●		●		●	●	
Sanger MRSA 252			●	●		●	●		●	●		●	●	●	●
ST75-MRSA-IV	○		●	●		○	○	○	●	○		●			
ST883-MRSA-IV	○		○			○	○		●				●	●	

● = positive in both amplification protocols

○ = positive in random amplification only
