# Supplementary material for: Characterisation of Australian MRSA Strains ST75- and ST883-MRSA-IV and Analysis of Their Accessory Gene Regulator Locus
Source: PLoS One. 2010 Nov 17;5(11):e14025. doi: 10.1371/journal.pone.0014025 (PMC2984443; doi:10.1371/journal.pone.0014025)
Supplement: File S3 — This files shows the detection of enterotoxin B in studied strains and reference strains. (2.78 MB PDF) [file pone.0014025.s003.pdf]

Supplemental File S3: Detection of enterotoxin B

Array layout [36], as diagramm (left) and with the image of isolate 05-15398 superimposed (right)

1	39	40	41	42	43	44	45	46	47	47	1
	28	29	30	31	32	33	34	35	36	37	38
1	17	18	19	20	21	22	23	24	25	26	27
1	6	7	8	9	10	11	12	13	14	15	16
39	40	41	42	43	44	45	46	2	3	4	5
27	28	29	30	31	32	33	34	35	36	37	38
15	16	17	18	19	20	21	22	23	24	25	26
3	4	5	6	7	8	9	10	11	12	13	14
36	37	38	39	40	41	42	43	44	45	46	2
24	25	26	27	28	29	30	31	32	33	34	35
12	13	14	15	16	17	18	19	20	21	22	23
1	2	3	4	5	6	7	8	9	10	11	1

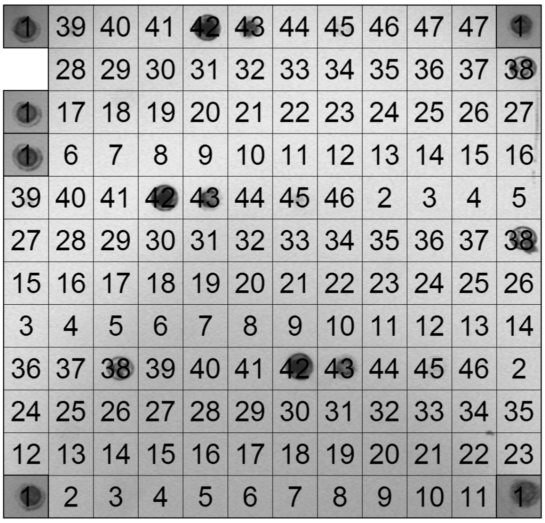

1 biotin marker (staining controls; their asymmetrical distribution allows the correct alignment of the array to the grid)

2 - 4 ricin specific antibodies

5 - 36 botulinum toxin specific antibodies

37 *Staphylococcus aureus* enterotoxin A specific antibody

38 *Staphylococcus aureus* enterotoxin B specific antibody
39 *Staphylococcus aureus* enterotoxin B specific antibody
40 *Staphylococcus aureus* enterotoxin B specific antibody
41 *Staphylococcus aureus* enterotoxin B specific antibody
42 *Staphylococcus aureus* enterotoxin B specific antibody
43 *Staphylococcus aureus* enterotoxin B specific antibody
44 *Staphylococcus aureus* enterotoxin B specific antibody

45 *Staphylococcus aureus* enterotoxin C specific antibody

46 *Staphylococcus aureus* enterotoxin A/B/C/D/E cross-reactive antibody

47 negative control (buffer)

**Sample / strain**	**Genotypic detection of *seb* by**		**Phenotypic detection of enterotoxin B by protein array**	
	**Primer-directed amplification**	**Random amplification**		
**Recombinant SEB, 0.01 µg/ml**<br>(as positive control)	n.a.	n.a.	POSITIVE	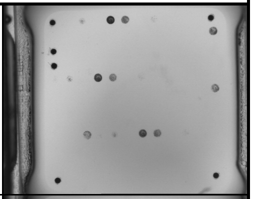
**ST250-MRSA-I, „Early/Ancestral MRSA“ reference strain COL**<br>(as positive control)	POSITIVE	POSITIVE	POSITIVE	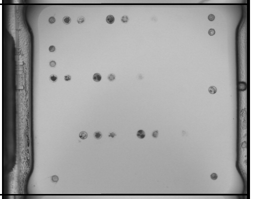
**CC8-MRSA-IV, „Lyon Clone/UK-EMRSA-2“ isolate 2006VA041111**<br>(as negative control)	NEGATIVE	n.a.	NEGATIVE	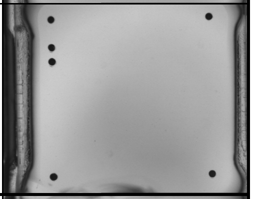
**ST75-MRSA-IV, isolate 03-17848**	NEGATIVE	AMBI-GUOUS	NEGATIVE	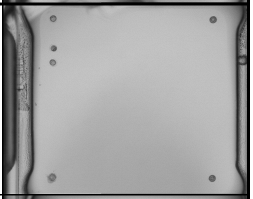
**ST75-MRSA-IV, isolate 04-16785**	NEGATIVE	AMBI-GUOUS	NEGATIVE	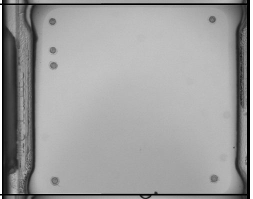
**ST75-MRSA-IV, isolate 04-17542**	POSITIVE	POSITIVE	POSITIVE	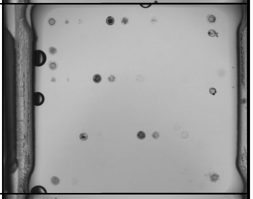
**ST75-MRSA-IV, isolate 05-15398**	POSITIVE	POSITIVE	POSITIVE	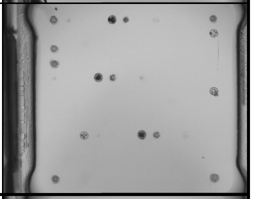
**ST883-MRSA-IV, isolate 06-16607**	NEGATIVE	NEGATIVE	NEGATIVE	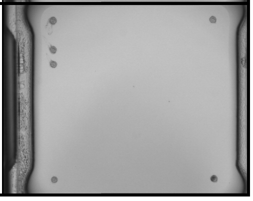
