# Supplementary material for: Characterisation of Australian MRSA Strains ST75- and ST883-MRSA-IV and Analysis of Their Accessory Gene Regulator Locus
Source: PLoS One. 2010 Nov 17;5(11):e14025. doi: 10.1371/journal.pone.0014025 (PMC2984443; doi:10.1371/journal.pone.0014025)
Supplement: File S4 — This file shows that, despite of unique agr gene sequences, predicted autoinducing peptide sequences of ST75 and ST883 allow them to be assigned to agr groups I and IV, respectively. (0.01 MB PDF) [file pone.0014025.s004.pdf]

Supplemental File S4: Alignment of putative amino acid sequences of AIPs from ST75, ST883 and from *agr* groups I to IV.

AIP-I:	------------------------YSTCDFIM---------------
NCTC8325, CP000253.1[2094440:2094580]:	MNTLFNLFFDFITGILKNIGNIAAYSTCDFIMDEVEVPKELTQLHE-
COL, CP000046.1[2083704:2083844]:	MNTLFNLFFDFITGILKNIGNIAAYSTCDFIMDEVEVPKELTQLHE-
FPR3757, CP000255.1[2147508:2147648]:	MNTLFNLFFDFITGILKNIGNIAAYSTCDFIMDEVEVPKELTQLHE-
UK9810618, DQ157966.1[2183:2323]:	MNTLFNLFFDFITGILKNIGNIAAYSTCDFIMDEVEVPKELTQLHE-
**ST75-MRSA-IV 03-17848, FJ154839.1[578:718]:**	**MNTLFNLLFELITGILKNIGNIAAYSTCDFIMDEVEVPKELTQLHE-**

AIP-II:	-----------------------GVNACSSLF---------------
Mu50, BA000017.4[2156442:2156585]:	MNTLVNMFFDFIIKLAKAIGIVGGVNACSSLFDEPKVPAELTNLYDK
N315, BA000018.3[2080012:2080155]:	MNTLVNMFFDFIIKLAKAIGIVGGVNACSSLFDEPKVPAELTNLYDK
RF122, AJ938182.1[2046158:2046301]:	MNTLVNMFFDFIIKLAKAIGIVGGVNACSSLFDEPKVPAELTNLYDK

AIP-III:	------------------------YINCDFLL---------------
MW2, BA000033.2[2108616:2108756]:	MKKLLNKVIELLVDFFNSIGYRAAYINCDFLLDEAEVPKELTQLHE-
MSSA476, BX571857.1[2087741:2087881]:	MKKLLNKVIELLVDFFNSIGYRAAYINCDFLLDEAEVPKELTQLHE-
MRSA252, BX571856.1[2184634:2184774]:	MKKLLNKVIELLVDFFNSIGYRAAYINCDFLLDEAEVPKELTQLHE-

AIP-IV:	------------------------YSTCYFIM---------------
H560, DQ157981.1[2182:2322]:	MNTLLNIFFDFITGVLKNIGNVASYSTCYFIMDEVEIPKELTQLHE-
RN4850, DQ229853.1[2198:2338]:	MNTLLNIFFDFITGVLKNIGNVASYSTCYFIMDEVEIPKELTQLHE-
**ST883-MRSA-IV 06-16607:**	**MNTLLNLFFNLITGILKNIGNVASYSTCYFIMDEVEIPKELTQLHE-**
